# Comparative transcriptomic analysis reveals the protective mechanism of ferulic acid against lipopolysaccharide-induced ferroptosis in IPEC-J2 cells

**DOI:** 10.3389/fvets.2026.1950560

**Published:** 2026-10-12

**Authors:** Jiayu Chen, Hongchao Li, Yaxuan Niu, Tao Guo, Xiangyu Wang, Yue Zhang, Wenwen Wang, Yuanqing Xu, Yuan Wang, Mingxing Chu

**Affiliations:** 1 College of Animal Science, Inner Mongolia Agricultural University, Hohhot, China; 2 Laboratory of Smart Animal Husbandry at Universities of Inner Mongolia Autonomous Region, Hohhot, China; 3 Dairy-Breeding and Production Research Subcenter of National Technology Innovation Center for Dairy, Hohhot, China; 4 State Key Laboratory of Animal Biotech Breeding, Institute of Animal Science, Chinese Academy of Agricultural Sciences, Beijing, China

**Keywords:** ferroptosis, ferulic acid, IPEC-J2, lipopolysaccharide, transcriptomic

## Abstract

Lipopolysaccharide (LPS) is a key virulence factor that induces intestinal oxidative damage in livestock and poultry, and cellular ferroptosis is one of the triggers of oxidative stress. Ferulic acid (FA) is a natural phenolic acid with antioxidant, anti-inflammatory and anti-ferroptosis properties. This study aimed to investigate the protective effects of FA against LPS-induced ferroptosis in the porcine small intestinal epithelial cell line (IPEC-J2) and to elucidate the underlying mechanisms. The experiment included four treatment groups: control group (CON), 50 μg/mL FA group (FA), 10 μg/mL LPS group (LPS), and 50 μg/mL FA combined with 10 μg/mL LPS group (FA and LPS), with all treatments lasting 24 h. The results showed that FA inhibited ferroptosis in IPEC-J2 cells. LPS successfully induced ferroptosis, as evidenced by decreased cell viability, increased levels of ROS, MDA, and Fe^2+^, downregulation of *GPX4* and *SLC7A11*, and upregulation of *TFRC* and *ACSL4*. FA effectively alleviated LPS-induced ferroptosis in IPEC-J2 cells, with an efficacy comparable to that of the ferroptosis inhibitor Fer-1. Transcriptomic analysis revealed that FA significantly reversed the gene expression changes induced by LPS, with differentially expressed genes enriched in pathways related to ferroptosis, TNF signaling, NF-κB signaling, arachidonic acid metabolism, and PI3K-Akt signaling. Protein–protein interaction (PPI) network analysis indicated that genes such as *HMGCS1*, *CXCL8*, and *IL-18* may serve as key nodes mediating the protective effects of FA. In conclusion, FA alleviates LPS-induced ferroptosis in IPEC-J2 cells by regulating glutathione metabolism, maintaining iron homeostasis, and inhibiting lipid peroxidation. This effect is closely associated with the coordinated regulation of inflammatory responses, lipid metabolism, and ferroptosis-related signaling pathways.

## 1 Introduction

The intestine is a key organ responsible for digestion, absorption, and immune defense in animals. Its structural integrity and functional homeostasis directly affect productive performance, health status, and product quality. It is also essential for the efficient and sustainable development of animal husbandry (1). In intensive farming systems, livestock and poultry often face environmental stress, nutritional imbalance, and pathogenic infections. These factors can induce intestinal stress and lead to intestinal injury (2). Gram-negative bacteria are common pathogens. Their cell wall component, lipopolysaccharide (LPS), is a major virulence factor that induces intestinal inflammation and damage (3). LPS can be recognized by intestinal epithelial cells. It activates downstream inflammatory signaling pathways. This process compromises epithelial barrier integrity by disrupting tight junctions, thereby increasing intestinal permeability. (4). To further investigate the molecular mechanisms of LPS-induced intestinal injury, this study used the porcine intestinal epithelial cell line IPEC-J2 as a classical model.

Previous studies have shown that LPS exposure can induce inflammatory cascades, oxidative stress, mitochondrial dysfunction, and disruption of tight junctions between cells (5). LPS binds to Toll-like receptor 4 (TLR4) on the surface of intestinal epithelial cells. This interaction activates downstream inflammatory signaling pathways, such as NF-κB. It also induces excessive production of reactive oxygen species (ROS), which leads to redox imbalance (6). Persistent oxidative stress and inflammation can further damage membrane lipids and proteins. They may also disrupt iron metabolism, which results in abnormal accumulation of intracellular ferrous ions (Fe^2+^). Recent studies have shown that enhanced lipid peroxidation, glutathione (GSH) depletion, and iron accumulation can trigger ferroptosis (7). Ferroptosis is a form of cell death that depends on iron and is driven by lipid peroxidation (8). In intestinal diseases, ferroptosis has been reported to participate in pathological processes such as ulcerative colitis and necrotizing enterocolitis. This suggests that it may play a key role in intestinal injury (9, 10). However, most existing studies focus on inflammatory bowel disease. The specific molecular mechanisms by which LPS induce intestinal epithelial cell injury through ferroptosis remain unclear. Therefore, it is important to systematically clarify the mechanisms of LPS-induced intestinal injury and to explore effective intervention strategies. This will help protect intestinal health in animals.

Extracts from traditional Chinese herbal medicine can reduce intracellular levels of reactive ROS and malondialdehyde (MDA). They can also increase the protein expression of ferritin and GPX4. These effects help reverse or alleviate ferroptosis in cells (11). Ferulic acid (FA) is a naturally occurring phenolic acid abundant in traditional Chinese medicinal herbs, including Angelica sinensis (Danggui) and Ligusticum chuanxiong (Chuanxiong), and exhibits a broad range of biological activities, including antioxidant, anti-inflammatory, and lipid metabolism-regulating effects. Our previous study showed that FA can alleviate LPS-induced ferroptosis in primary sheep hepatocytes (12). However, the effects of FA on ferroptosis in intestinal epithelial cells remain unclear. The protective role of FA against LPS-induced ferroptosis in IPEC-J2 cells has not been systematically studied. Therefore, this study used IPEC-J2 cells as a model to investigate the mechanism by which FA alleviates LPS-induced ferroptosis. This work provides a theoretical basis for the use of FA as a potential feed additive targeting ferroptosis to maintain intestinal health in livestock and poultry.

## 2 Materials and methods

### 2.1 Cell culture and treatments

The IPEC-J2 cell line was obtained from Beina Bio (China). This cell line originates from porcine jejunal epithelium. It is a well-established *in vitro* model for studying intestinal function in pigs, including barrier integrity, immune response, and nutrient absorption. Cells were cultured in DMEM/F12 medium (Gibco, USA). The medium was supplemented with 10% fetal bovine serum (FBS, Gibco), 100 U/mL penicillin, and 100 μg/mL streptomycin. Cultures were maintained at 37 °C in a humidified incubator with 5% CO₂. Cells were passaged every 2 to 3 days. When cultures reached 80–90% confluency, cells were dissociated using 0.25% trypsin–EDTA (Gibco, USA). All experiments used cells in the logarithmic growth phase under optimal conditions.

### 2.2 Reagent preparation and experimental groups

FA (Pengyu Biotechnology Co., China) solution: A stock solution of 10 mg/mL FA was prepared in PBS (Gibco, USA) and stored for subsequent use. LPS solution: A stock solution of 5 mg/mL LPS was prepared in PBS and stored for subsequent use. Ferrostatin-1 (Fer-1, MCE, USA) solution: A stock solution of 10 μmol/L Fer-1 was prepared in DMSO (Gibco, USA) and stored for subsequent use. All stock solutions were aliquoted and stored at −20 °C. Immediately before each experiment, working solutions were freshly prepared by diluting the stocks in PBS (FA and LPS) or in complete DMEM/F12 medium (Fer-1). IPEC-J2 cells were seeded in appropriate culture plates and allowed to adhere overnight prior to the following treatments: (1) FA treatment: Cells were treated with FA at 0, 1, 10, 100, 500, or 1,000 μg/mL for 24 h. (2) LPS treatment: Cells were treated with LPS at 0, 1, 10, 50, 100, or 200 μg/mL for 24 h. (3) Fer-1 concentration screening: Cells were treated for 24 h as follows: control (CON); LPS (10 μg/mL); or LPS (10 μg/mL) combined with Fer-1 at 2, 4, 6, 8, or 10 μmol/L. (4) FA concentration screening: Cells were treated for 24 h as follows: CON; LPS (10 μg/mL); or LPS (10 μg/mL) combined with FA at 50, 100, 200, or 400 μg/mL. (5) Assessment of FA’s protective effect: Cells were treated for 24 h in one of six groups: CON; LPS (10 μg/mL); FA (50 μg/mL); Fer-1 (4 μmol/L); LPS and FA (10 μg/mL and 50 μg/mL); or LPS and Fer-1 (10 μg/mL and 4 μmol/L). (6) RNA sequencing and validation: Cells were treated for 24 h in one of four groups: CON; FA (50 μg/mL); LPS (10 μg/mL); or FA and LPS (50 μg/mL and 10 μg/mL). For all *in vitro* experiments, three independent biological replicates were performed, each with three technical replicates.

### 2.3 Cellular assays

Cells were seeded into 96-well plates at a density of 1 × 10^5^ cells/mL (200 μL per well) and pre-cultured for 12 h. Following treatment according to the experimental design, 10 μL of CCK-8 solution (Dojindo, Japan) was added to each well. After incubation for 2 h at 37 °C in the dark, the absorbance at 450 nm was measured using a microplate reader. Cell viability was expressed as a percentage of the control group’s OD value. The levels of reactive ROS, MDA, superoxide dismutase (SOD), total antioxidant capacity (T-AOC), catalase (CAT), Fe^2+^, and the ratio of reduced to Glutathione/Glutathione disulfide (GSH/GSSG) were measured using commercial assay kits according to the respective manufacturers’ protocols. Kits for MDA, SOD, T-AOC, CAT, and Fe^2+^ were purchased from Nanjing Jiancheng Bioengineering Institute (China). Kits for ROS, GSH, and GSSG were obtained from Beyotime Biotechnology (China).

### 2.4 qRT-PCR

IPEC-J2 cells were seeded into 6-cm dishes at a density of 1 × 10^6^ cells/mL (5 mL per dish) and cultured for 12 h. Processed samples were snap-frozen immediately in liquid nitrogen and subsequently stored at −80 °C for total RNA extraction. Total RNA was extracted from IPEC-J2 cells using a total RNA extraction kit (Invitrogen, USA). RNA concentration and purity were assessed with a microvolume spectrophotometer, and A260/A280 ratios ranged between 1.8 and 2.1. RNA integrity was verified by 1.5% agarose gel electrophoresis. For cDNA synthesis, 1 μg of total RNA was subjected to genomic DNA removal and reverse transcription according to the protocol of the PrimeScript™ RT reagent Kit (TaKaRa, Japan). Reverse transcription was performed at 37 °C for 15 min, followed by 85 °C for 5 s. The resulting cDNA was stored at −20 °C until further analysis. Real-time quantitative PCR was conducted on a QuantStudio 5 Real-Time PCR System using TB Green® Premix Ex Taq™ (TaKaRa, Japan). The thermal cycling conditions were as follows: initial denaturation at 95 °C for 30 s, followed by 40 cycles of 95 °C for 5 s and 60 °C for 30 s. β-actin served as the internal reference gene, and the relative mRNA expression levels of target genes were calculated using the 2^-ΔΔCt^ method (13). Each sample was analyzed with three technical replicates, and the entire experiment was independently repeated three times. The primer sequences are listed in Table 1.

**Table 1:** Primer sequence used for quantitative qRT-PCR.

Gene	Nucleotide Sequences 5′ to 3′	Size/bp
ABCG1	F: GTGTACTGGATGACGTCGCA	199
	R: CGAAGCTGACGAAGAACCCT	
CXCL8	F: ACGCATTCCACACCTTTCCAC	157
	R: TCTGTACAACCTTCTGCACCC	
LPCAT3	F: TCACTGGTACCATCGCCTCT	222
	R: TGTCTTGAATCAGGCTGGCA	
IL6	F: AGAATCACCACCGGTCTTGT	95
	R: ATCTGCACAGCCTCGACATT	
PFKF83	F: GAATGTGGAGTCGGTGAGCA	225
	R: CCTTTCACGTTTTGTCCGGG	
SCD	F: GCCACCTTTCTTCGTTACGC	142
	R: CCTCACCCACAGCTCCCAAT	
ALOX5AP	F: TCATCAGCGTGGTCCAGAAC	147
	R: TGGGGTATGCATCGACACAG	
TAGLN	F: ATAGTGCAGTGTGGCCCTG	200
	R: GCGGCCTTTAAGAACTGAGC	
ACSL4	F: TTGCCAAGAGTGACCAGTCC	215
	R: TGGGCTTAAGCGAACCTTGA	
GPX4	F: TTACGGATTCTGGCCTTCCC	286
	R: CTATGACCTGGGGCTCTTCC	
TFRC	F: ATGGACCGGGCTAACTTTCC	94
	R: GGAATCCAGGTGTGTACGGG	
SLC7A11	F: CTGGGCAGGAGAAAGTTGT	285
	R: AGACTCGCACAAAAGCTGGT	

### 2.5 RNA extraction, library construction, and sequencing

IPEC-J2 cells (1 × 10^6^ cells/mL, 5 mL) were seeded into 6 cm culture dishes and incubated at 37 °C in a 5% CO₂ incubator for 12 h. Once the cells reached 60–70% confluence, they were divided into groups. Based on the optimal dosage determined in previous experiments, four treatment groups were established: control group (CON), FA group (50 μg/mL FA), LPS group (10 μg/mL LPS), and FA and LPS group (50 μg/mL FA and 10 μg/mL LPS). All treatments were performed for 24 h, with three biological replicates per group. After treatment, the cells were washed twice with PBS and collected. Total RNA was extracted using Trizol (Invitrogen, USA) following the manufacturer’s instructions. RNA integrity was assessed using an Agilent 2,100 Bioanalyzer (Agilent Technologies, USA), with a requirement for the RNA Integrity Number (RIN) to be >7.0. The RNA integrity was further verified by agarose gel electrophoresis. RNA samples that passed the integrity check were sent to Shanghai Meiji Biological Pharmaceutical Technology Co., Ltd. (China) for library construction and sequencing. Library construction followed the Illumina platform’s standard procedures: mRNA was enriched and purified using Oligo(dT) magnetic beads, while rRNA and other non-coding RNAs were removed. The mRNA was fragmented into approximately 300 bp segments using Mg^2+^ ion fragmentation buffer to ensure uniform fragment length. First-strand cDNA was synthesized by reverse transcription using 6 bp random primers, and second-strand cDNA was synthesized using the first-strand cDNA as a template and dNTPs. The cDNA product was purified using a PCR purification kit (Qiagen, Venlo, The Netherlands), followed by end repair, poly(A) tail addition, and Illumina sequencing adapter ligation. The final sequencing library was assessed for size using the Agilent 2,100 Bioanalyzer and proceeded to sequencing upon passing size qualification. Sequencing was carried out on the Illumina NovaSeq 6,000 platform (Illumina, USA) with a 150 bp paired-end read strategy (PE 150 bp). The sequencing process adhered strictly to the platform’s standard operating procedures, with sequencing quality controlled to ensure a Q30 value ≥85%, ensuring the accuracy of the raw sequencing data. Each sample generated at least 6 Gb of sequencing data to meet the requirements for subsequent bioinformatics analysis. The final raw sequencing data (raw reads) were used for further data analysis.

### 2.6 RNA sequencing data analysis

First, rigorous quality control (QC) was performed on the raw sequencing data (Raw reads). Base quality, adapter contamination, and the proportion of N bases were assessed using FastQC software (v0.11.9). Low-quality data were filtered with Trimmomatic software (v0.39). Reads containing adapter sequences, low-quality reads (with more than 50% bases having a quality score <20), and reads with more than 10% N bases were removed. High-quality clean reads were obtained. After quality control, clean reads were rechecked with FastQC software to ensure no significant adapter remnants and that the base quality met the required standards. Next, sequence alignment and gene expression quantification were performed. The clean reads were aligned to the pig reference genome (*Sus scrofa* 11.1) using HISAT2 software (v2.2.1). Default alignment parameters were used to ensure efficiency and accuracy, with an alignment rate of ≥85%. Gene expression was quantified by counting the number of reads mapped to each gene using HTSeq software (v0.11.2). FPKM (Fragments Per Kilobase of transcript per Million mapped reads) was used to quantify gene expression. A higher FPKM value indicates higher gene expression. Finally, differential gene expression analysis was performed. DESeq2 software (v1.34.0) was used to analyze gene expression differences between the groups. Differentially expressed genes (DEGs) were identified using a raw *p*-value threshold of < 0.05.

### 2.7 KEGG pathway enrichment analysis

To further explore the potential biological functions of the candidate DEGs identified under different treatments, Kyoto Encyclopedia of Genes and Genomes (KEGG) pathway enrichment analysis was performed using KOBAS software (v3.0) based on the hypergeometric test. Candidate DEGs identified using a nominal *p*-value < 0.05 were used as input for the enrichment analysis. Enrichment *p*-values were calculated for individual KEGG pathways, and *p* < 0.05 was considered statistically significant. In addition to statistically significant pathways, selected pathways closely related to the biological processes investigated in this study, including ferroptosis, lipid metabolism, oxidative stress, and inflammatory responses, were retained for exploratory visualization and interpretation. Pathways that did not meet the statistical significance threshold were not interpreted as significantly enriched.

### 2.8 PPI network construction and module analysis

To reveal the functional associations between proteins encoded by DEGs at the system level, this study further constructed a protein–protein interaction (PPI) network. The PPI network was constructed using the STRING online database (version 11.0, https://string-db.org/), with *Sus scrofa* as the species. Interaction relationships with a combined confidence score (combined_score) ≥ 400 were retained to control the network quality. The PPI network was then visualized using Cytoscape software (v3.6.1, http://cytoscape.org/). Topological parameters, including degree centrality and betweenness centrality, were calculated to identify hub genes in the network. These genes typically play a central regulatory role in functional modules. To screen for hub genes and significant network modules, we used the Cytoscape plugins cytoHubba and Molecular Complex Detection (MCODE). Hub genes were selected using the maximum clique centrality (MCC) method, and significant network modules were identified using the default parameters of the MCODE plugin.

### 2.9 Statistical analysis

The raw experimental data were organized and calculated using Excel 2010. Normality and homogeneity of variance were assessed before ANOVA. The data were then analyzed by one-way analysis of variance (ANOVA) using SAS 9.2 software. Multiple comparisons were performed using Tukey’s test. Statistical graphs were generated using GraphPad Prism 9.0 software. Differences were considered significant at *p* ≤ 0.05.

## 3 Results

### 3.1 Effects of FA on cell viability, antioxidant capacity and Iron metabolism in IPEC-J2 cells

To clarify the effect of FA treatment alone on IPEC-J2 cells, as shown in Figure 1, compared with the control group, supplementation with 1–500 μg/mL FA significantly increased the cell proliferation rate (*p* < 0.05) and significantly reduced the relative fluorescence intensity of ROS (*p* < 0.05). Supplementation with 1–100 μg/mL FA significantly increased CAT activity (*p* < 0.05) and significantly decreased MDA content (*p* < 0.05). Supplementation with 1 and 100 μg/mL FA significantly increased T-AOC content (*p* < 0.05). Supplementation with 10 and 100 μg/mL FA significantly increased the GSH/GSSG ratio (*p* < 0.05). Supplementation with 1–100 μg/mL FA had no significant effect on SOD activity. Supplementation with 100 and 500 μg/mL FA significantly reduced Fe^2+^ content (*p* < 0.05). Notably, compared with the control group (0 μg/mL FA), supplementation with 1,000 μg/mL FA significantly decreased CAT and SOD activities, T-AOC content, and the GSH/GSSG ratio (*p* < 0.05), indicating that 1,000 μg/mL FA exhibited certain cytotoxicity. In summary, FA inhibited ferroptosis in IPEC-J2 cells, and 10–500 μg/mL was identified as the safe and effective concentration range.

**Figure 1:**
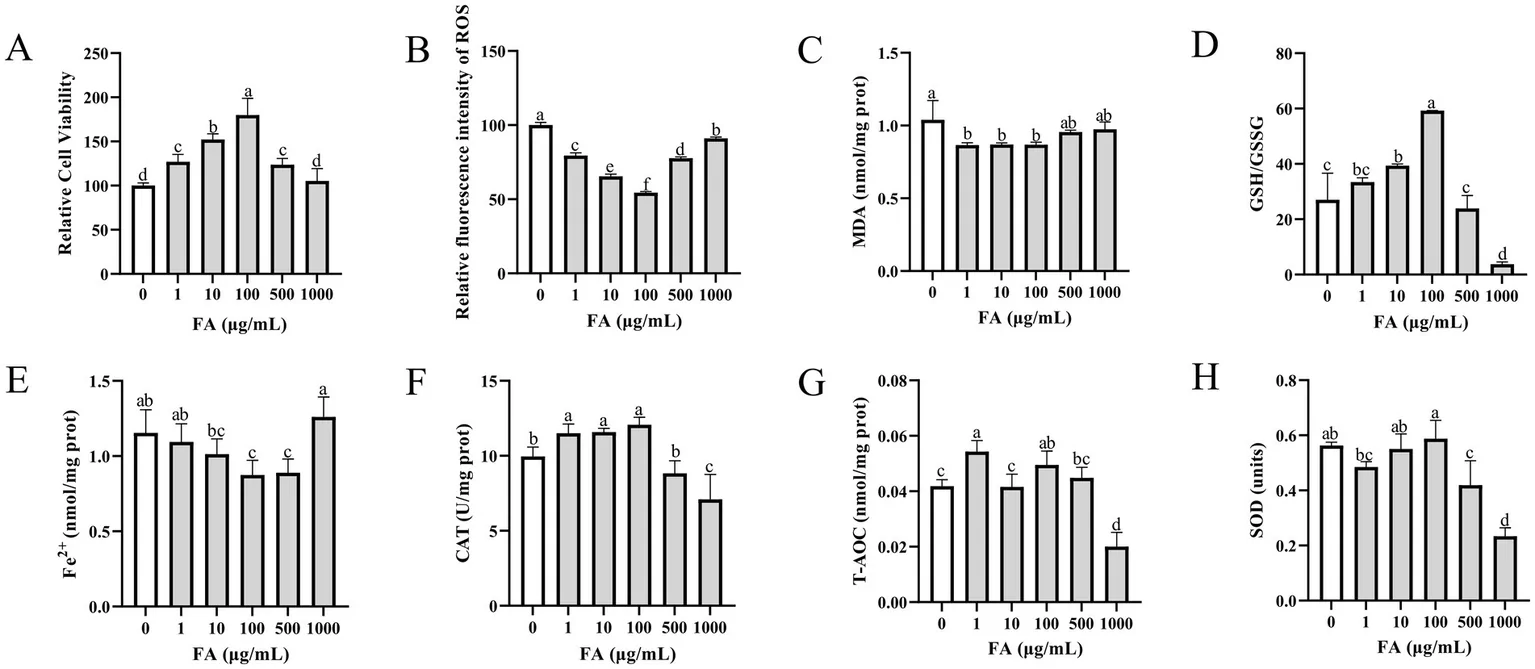
Effects of FA on Cell Viability, Antioxidant Capacity and Iron Metabolism in IPEC-J2 Cells. **(A)** Effects of different concentrations of FA on cell viability; **(B)** effects of different concentrations of FA on ROS; **(C)** effects of different concentrations of FA on MDA; **(D)** effects of different concentrations of FA on the GSH/GSSG ratio; **(E)** effects of different concentrations of FA on Fe^2+^; **(F)** effects of different concentrations of FA on CAT; **(G)** effects of different concentrations of FA on T-AOC; **(H)** effects of different concentrations of FA on SOD. Bars with the same letters or without letters indicate no significant difference (*p* > 0.05), whereas bars with different lowercase letters indicate significant differences (*p* < 0.05). The same applies to the following figures.

### 3.2 Effects of LPS on cell viability, oxidative damage and Iron metabolism in IPEC-J2 cells

To clarify the mechanism by which LPS induces injury in IPEC-J2 cells, as shown in Figure 2, compared with the control group (0 μg/mL LPS), supplementation with 1–200 μg/mL LPS significantly reduced cell viability (*p* < 0.05). At the same time, it significantly increased the relative fluorescence intensity of ROS, as well as MDA and Fe^2+^ contents (*p* < 0.05). These results indicate that LPS successfully induced ferroptosis in IPEC-J2 cells. Among the tested concentrations, 10 μg/mL LPS caused moderate cell damage with obvious ferroptosis-related phenotypic changes, while avoiding excessive cell death that would interfere with subsequent drug-intervention assays. Based on the extent of cell injury and the feasibility of subsequent experiments, 10 μg/mL LPS was selected as the optimal concentration to induce ferroptosis in IPEC-J2 cells.

**Figure 2:**
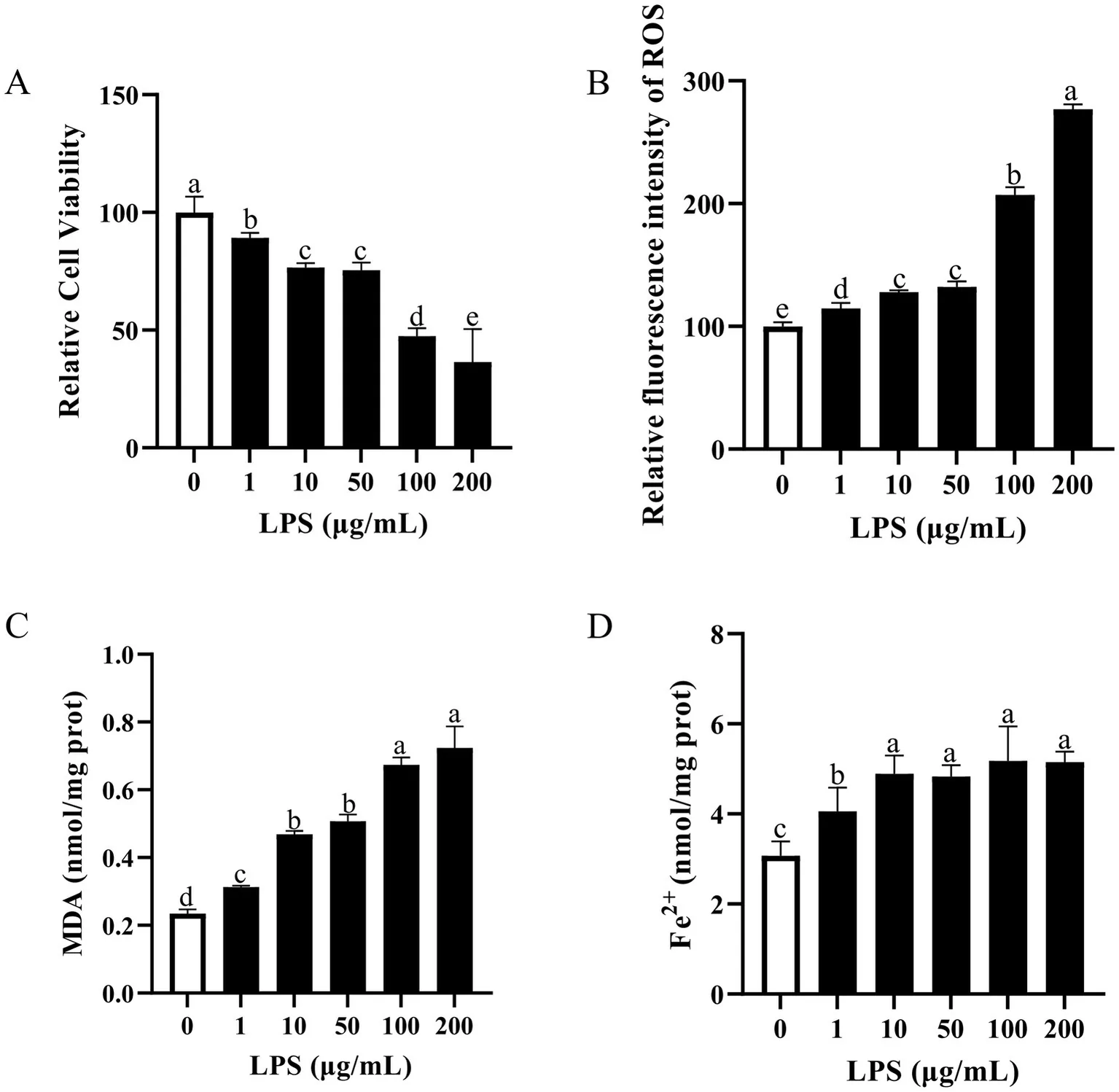
Effects of LPS on Cell Viability, Oxidative Damage and Fe^2+^ in IPEC-J2 Cells. **(A)** Effects of different concentrations of LPS on cell viability; **(B)** effects of different concentrations of LPS on ROS; **(C)** effects of different concentrations of LPS on MDA; **(D)** effects of different concentrations of LPS on Fe^2+^.

### 3.3 FA alleviates LPS-induced ferroptosis in IPEC-J2 cells

To determine the protective effect of FA on LPS-induced ferroptosis in IPEC-J2 cells, as shown in Figure 3, compared with the LPS group, the LPS and FA50 group and LPS and FA100 group significantly increased cell viability (*p* < 0.05). They also significantly reduced the relative fluorescence intensity of ROS, as well as MDA and Fe^2+^ contents (*p* < 0.05). These results indicate that supplementation with 50 and 100 μg/mL FA can alleviate LPS-induced ferroptosis. Based on the degree of cell recovery and the feasibility of subsequent experiments, 50 μg/mL FA was selected as the optimal concentration for further studies.

**Figure 3:**
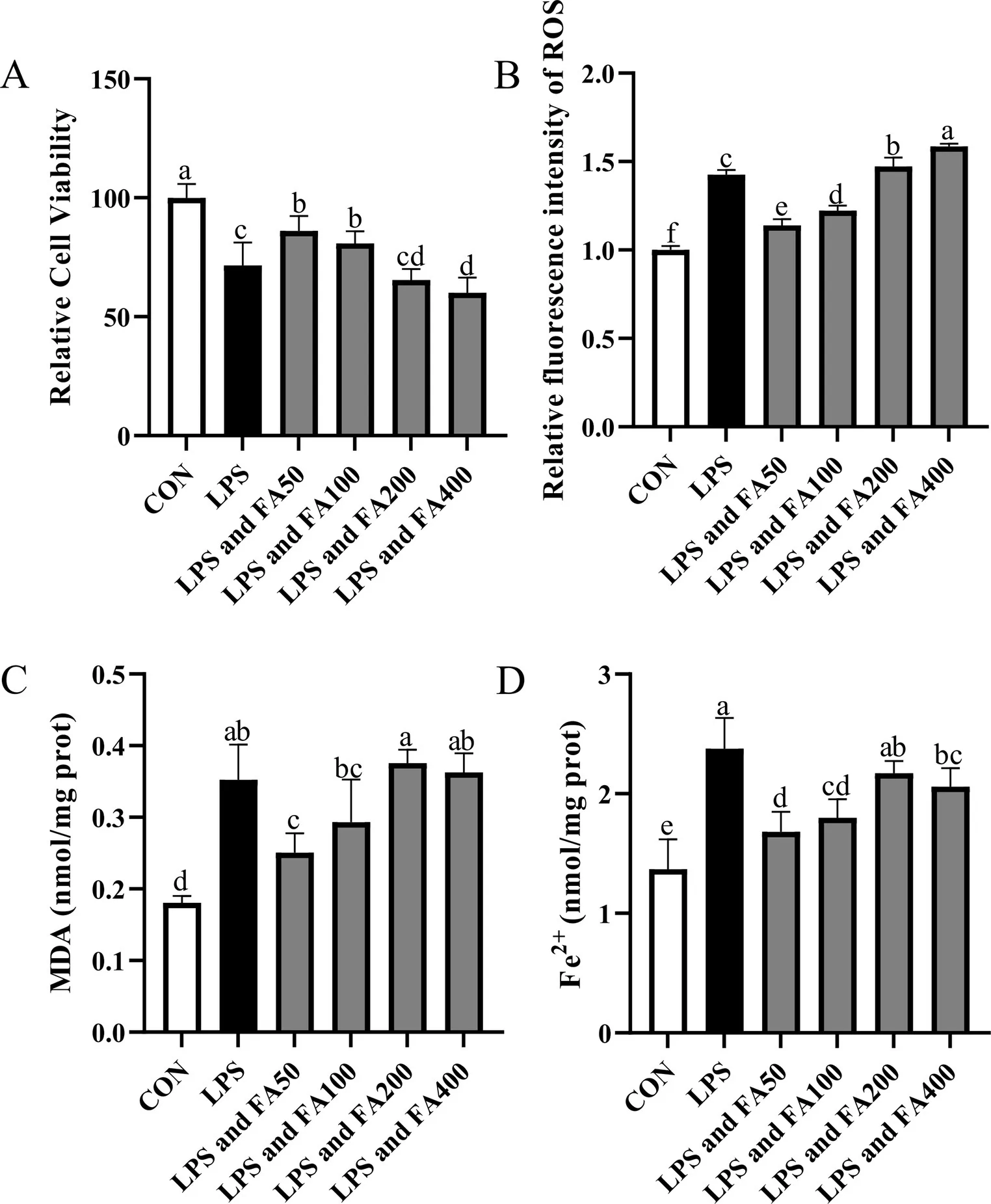
Effects of different concentrations of FA on LPS-induced ferroptosis in IPEC-J2 cells. **(A)** Effects of different concentrations of FA on cell viability under LPS stimulation; **(B)** effects of different concentrations of FA on ROS under LPS stimulation; **(C)** effects of different concentrations of FA on MDA under LPS stimulation; **(D)** effects of different concentrations of FA on Fe^2+^ under LPS stimulation. CON group: control group; LPS group: 10 μg/mL LPS; LPS and FA50 group: 10 μg/mL LPS + 50 μg/mL FA; LPS and FA100 group: 10 μg/mL LPS + 100 μg/mL FA; LPS and FA200 group: 10 μg/mL LPS + 200 μg/mL FA; LPS and FA400 group: 10 μg/mL LPS + 400 μg/mL FA.

To further investigate how FA protects IPEC-J2 cells by regulating ferroptosis, Fer-1 was introduced for validation. As shown in Figure 4, compared with the LPS group, both the LPS and FA group and the LPS and Fer-1 group significantly reduced MDA content, ROS relative fluorescence intensity, and Fe^2+^ levels (*p* < 0.05). Meanwhile, they significantly increased the GSH/GSSG ratio, CAT and SOD activities, and T-AOC content (*p* < 0.05). These findings indicate that FA alleviates LPS-induced ferroptosis in IPEC-J2 cells by enhancing antioxidant capacity, maintaining iron homeostasis, and regulating glutathione metabolism. This effect is consistent with the ferroptosis-inhibitory activity of Fer-1. As shown in Figure 5, compared with the LPS group, the FA group, Fer-1 group, and LPS and Fer-1 group significantly upregulated the relative mRNA expression level of *GPX4* (*p* < 0.05). The FA group, Fer-1 group, LPS and Fer-1 group, and LPS and FA group significantly upregulated the relative mRNA expression level of *SLC7A11* (*p* < 0.05). In addition, the FA group, Fer-1 group, LPS and Fer-1 group, and LPS and FA group significantly downregulated the relative mRNA expression levels of *TFRC* and *ACSL4* (*p* < 0.05). These results at the gene level indicate that LPS can induce ferroptosis in cells. Using Fer-1 as a positive control, the findings further demonstrate that FA can alleviate LPS-induced ferroptosis in IPEC-J2 cells.

**Figure 4:**
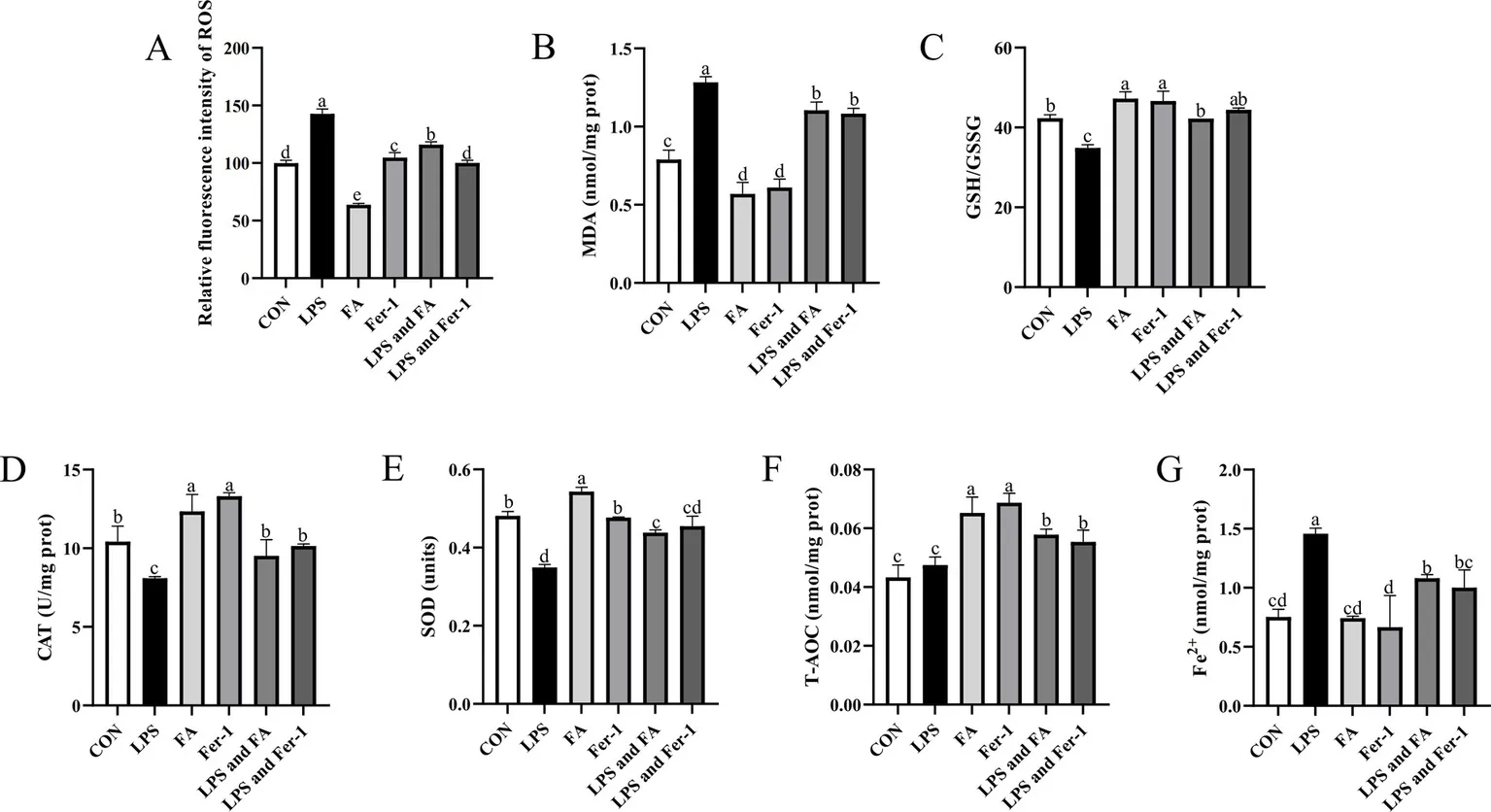
Effects of FA on LPS-induced ferroptosis in IPEC-J2 cells. **(A–G)** Effects of different treatments on ROS relative fluorescence intensity, MDA content, GSH/GSSG ratio, CAT content, SOD activity, T-AOC content, and Fe^2+^ content in IPEC-J2 cells. CON group: control group; LPS group: 10 μg/mL LPS; FA group: 50 μg/mL FA; Fer-1 group: 4 μmol/L Fer-1; LPS and FA group: 10 μg/mL LPS + 50 μg/mL FA; LPS and Fer-1 group: 10 μg/mL LPS + 4 μmol/L Fer-1.

**Figure 5:**
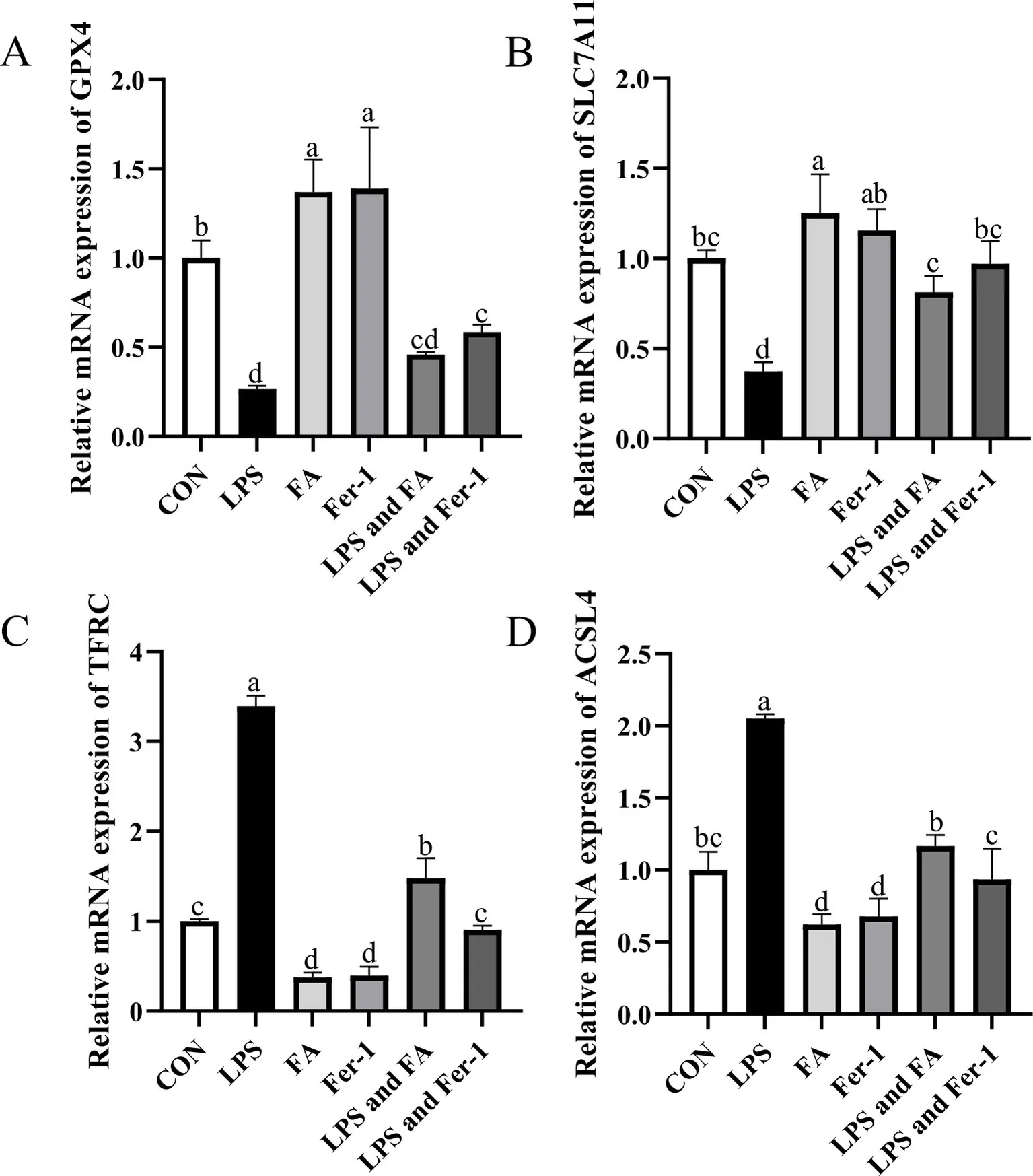
Effects of FA on ferroptosis-related marker genes in LPS-induced IPEC-J2 cells. **(A–D)** Effects of different treatments on the relative mRNA expression levels of GPX4, SLC7A11, TFRC, and ACSL4 in IPEC-J2 cells. CON group: control group; LPS group: 10 μg/mL LPS; FA group: 50 μg/mL FA; Fer-1 group: 4 μmol/L Fer-1; LPS and FA group: 10 μg/mL LPS + 50 μg/mL FA; LPS and Fer-1 group: 10 μg/mL LPS + 4 μmol/L Fer-1.

### 3.4 Comparative transcriptomics reveals the mechanism by which FA alleviates LPS-induced ferroptosis in IPEC-J2 cells

To clarify the molecular mechanisms by which FA alleviates LPS-induced intestinal epithelial cell injury through regulation of the ferroptosis pathway, RNA sequencing was performed in four groups: CON, LPS, FA, and FA and LPS. The sequencing quality was high. Q20 (base error rate <1%) and Q30 (base error rate <0.1%) were 99.23 and 96.19%, respectively. The overall base error rate was as low as 0.0118%. After removing reads containing poly-N sequences and adapter contamination, and filtering low-quality reads, about 41.51 million clean reads (99.34%) were obtained. The rRNA contamination rate was 0.773%, and the average GC content was 51.10%. These results indicate that the data met the requirements for high-quality sequencing analysis.

An expression matrix containing 35,682 genes was obtained from RNA sequencing. Compared with the CON group, the LPS group was used to establish a ferroptosis model. A total of 402 DEGs were identified, including 193 upregulated and 209 downregulated genes. Compared with the CON group, the FA group was used to evaluate the effect of ferulic acid alone on ferroptosis, and 2,799 DEGs were identified, including 1,350 upregulated and 1,449 downregulated genes. Compared with the CON group, the FA and LPS group was used to clarify the protective effects of FA through intervention in the above injury pathways. A total of 3,126 DEGs were identified, including 1,593 upregulated and 1,533 downregulated genes. Compared with the LPS group, the FA and LPS group was used to determine whether FA could specifically antagonize LPS-induced ferroptosis in IPEC-J2 cells by targeting core ferroptosis pathways and key gene expression. A total of 2,882 DEGs were identified, including 1,449 upregulated and 1,433 downregulated genes. Figure 6A shows the upregulated and downregulated genes in the comparisons of CON vs. LPS, CON vs. FA, CON vs. FA and LPS, and LPS vs. FA and LPS. In addition, co-expressed DEGs among CON vs. LPS, CON vs. FA and LPS, LPS vs. FA and LPS, and CON vs. FA were analyzed. As shown in the Venn diagram (Figure 6B), 162 DEGs were shared between CON vs. LPS and LPS vs. FA and LPS. Notably, 29 DEGs that were downregulated in CON vs. LPS were significantly upregulated in LPS vs. FA and LPS. In addition, 41 DEGs that were upregulated in CON vs. LPS were also significantly upregulated in LPS vs. FA and LPS (Table 2).

**Figure 6:**
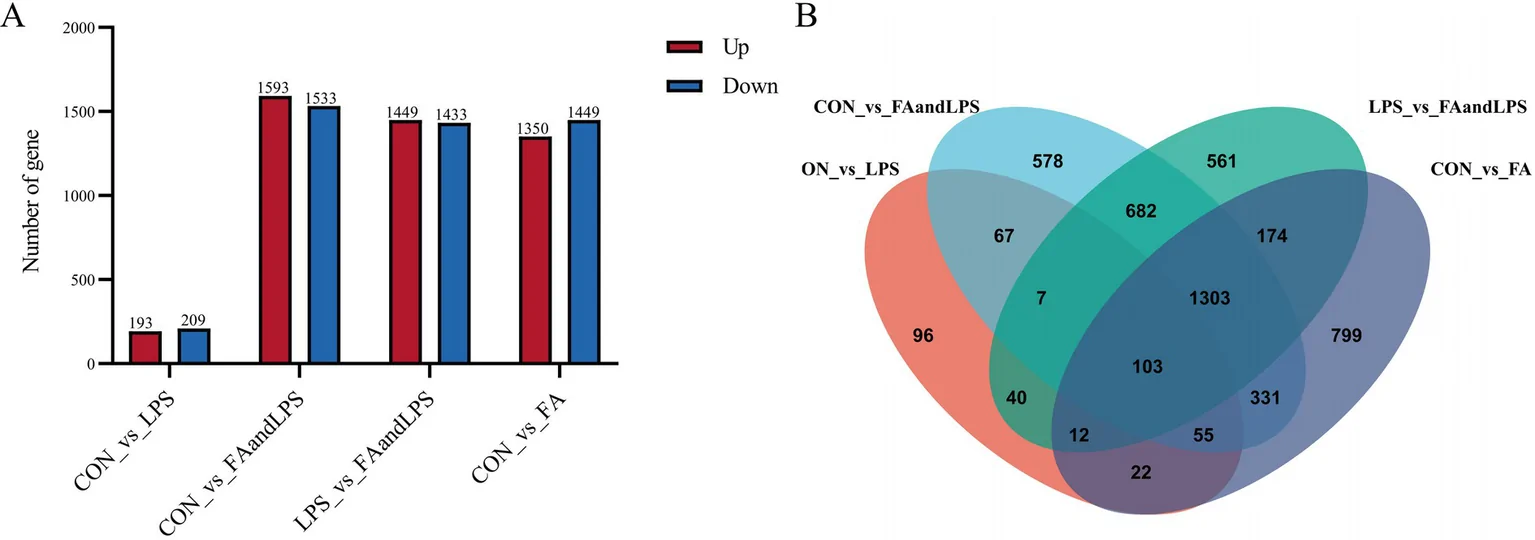
Differentially expressed gene (DEG) profiling based on RNA-seq analysis. **(A)** Number of upregulated and downregulated DEGs among different treatment group comparisons; **(B)** Venn diagram of DEGs. CON group: control group; FA group: 50 μg/mL FA; FA and LPS group: 10 μg/mL LPS + 50 μg/mL FA; LPS group: 10 μg/mL LPS.

**Table 2:** Summary of co-expressed differentially expressed genes between the CON vs. LPS and LPS vs. LPS + (and) FA groups.

Gene ID	Gene name	Full names of gens	CON vs. LPS	CON vs. FA	CON vs. LPS and FA	LPS vs. LPS and FA
ENSSSCG00000027745	ABCG1	Atp binding cassette subfamily g member 1	UP	Down	Up	DOWN
ENSSSCG00000004597	AQP9	Aquaporin 9	UP	Up	Down	DOWN
ENSSSCG00000016185	ARPC2	Actin related protein 2/3 complex subunit 2	UP	Down	Down	DOWN
ENSSSCG00000018080	ATP8	Atp synthase f0 subunit 8	UP	Down	Up	DOWN
ENSSSCG00000036136	BHLHE40	Basic helix–loop–helix family member e40	UP	Down	Up	DOWN
ENSSSCG00000040779	CBS	Cystathionine beta-synthase	UP	Down	Up	DOWN
ENSSSCG00000021259	CDA	Cytidine deaminase	UP	Down	Down	DOWN
ENSSSCG00000008094	CHCHD5	Coiled-coil-helix-coiled-coil-helix domain containing 5	UP	Down	Down	DOWN
ENSSSCG00000014924	CTSC	Cathepsin c	UP	Down	Down	DOWN
ENSSSCG00000008953	CXCL8	C-x-c motif chemokine ligand 8	UP	Down	Up	DOWN
ENSSSCG00000011628	DNAJC13	Dnaj heat shock protein family (hsp40) member c13	UP	Down	Down	DOWN
ENSSSCG00000013147	FAM111B	Fam111 trypsin like peptidase b	UP	Up	Up	DOWN
ENSSSCG00000052947	GSTA1	Glutathione s-transferase alpha 1	UP	Down	Down	DOWN
ENSSSCG00000039882	HTRA2	Htra serine peptidase 2	UP	Down	Up	DOWN
ENSSSCG00000027607	IER3	Immediate early response 3	UP	Down	Down	DOWN
ENSSSCG00000020970	IL6	Interleukin 6	UP	Up	Down	DOWN
ENSSSCG00000027205	LPCAT3	Lysophosphatidylcholine acyltransferase 3	UP	Down	Down	DOWN
ENSSSCG00000029698	LRRC42	Leucine rich repeat containing 42	UP	Down	Down	DOWN
ENSSSCG00000026981	MRPS6	Mitochondrial ribosomal protein s6	UP	Down	Down	DOWN
ENSSSCG00000003775	MSH4	Rab geranylgeranyltransferase subunit beta	UP	Down	Down	DOWN
ENSSSCG00000000859	PARPBP	Parp1 binding protein	UP	Up	Down	DOWN
ENSSSCG00000016884	PELO	Pelota mrna surveillance and ribosome rescue factor	UP	Down	Down	DOWN
ENSSSCG00000011133	PFKFB3	6-phosphofructo-2-kinase/fructose-2,6-biphosphatase 3	UP	Down	Down	DOWN
ENSSSCG00000012440	PGK1	Phosphoglycerate kinase 1	UP	Down	Down	DOWN
ENSSSCG00000002747	PMFBP1	Polyamine modulated factor 1 binding protein 1	UP	Up	Down	DOWN
ENSSSCG00000014406	PRELID2	Preli domain containing 2	UP	Down	Down	DOWN
ENSSSCG00000036658	PSMD8	Prostaglandin e synthase	UP	Down	Down	DOWN
ENSSSCG00000005688	PTGES	Prostaglandin e synthase	UP	Down	Down	DOWN
ENSSSCG00000010479	RBP4	Retinol binding protein 4	UP	Down	Up	DOWN
ENSSSCG00000036311	RPAIN	Rpa interacting protein	UP	Down	Down	DOWN
ENSSSCG00000010554	SCD	Stearoyl-coa desaturase	UP	Down	Down	DOWN
ENSSSCG00000042767	SERGEF	Secretion regulating guanine nucleotide exchange factor	UP	Down	Down	DOWN
ENSSSCG00000021443	SGK1	Serum/glucocorticoid regulated kinase 1	UP	Up	Down	DOWN
ENSSSCG00000017925	SLC16A11	Solute carrier family 16 member 11	UP	Down	Up	DOWN
ENSSSCG00000021968	SLC19A3	Solute carrier family 19 member 3	UP	Down	Down	DOWN
ENSSSCG00000031954	SLC39A10	Solute carrier family 39 member 10	UP	Down	Down	DOWN
ENSSSCG00000034167	SLC5A3	Solute carrier family 5 member 3	UP	Down	Down	DOWN
ENSSSCG00000014818	STARD10	Star related lipid transfer domain containing 10	UP	Down	Down	DOWN
ENSSSCG00000062098	TMEM218	Transmembrane protein 218	UP	Up	Up	DOWN
ENSSSCG00000026695	TNFSF14	Tnf superfamily member 14	UP	Down	Down	DOWN
ENSSSCG00000030388	UPP1	Uridine phosphorylase 1	UP	Down	Down	DOWN
ENSSSCG00000011782	ABCC5	Atp binding cassette subfamily c member 5	DOWN	Up	Up	UP
ENSSSCG00000001725	ADGRF5	Adhesion g protein-coupled receptor f5	DOWN	Up	Up	UP
ENSSSCG00000009330	ALOX5AP	Arachidonate 5-lipoxygenase activating protein	DOWN	Up	Down	UP
ENSSSCG00000024569	ANO9	Anoctamin 9	DOWN	Up	Up	UP
ENSSSCG00000035565	CCDC134	Coiled-coil domain containing 134	DOWN	Down	Up	UP
ENSSSCG00000004484	COL12A1	Collagen type xii alpha 1 chain	DOWN	Up	Up	UP
ENSSSCG00000004441	DSE	Dermatan sulfate epimerase	DOWN	Up	Down	UP
ENSSSCG00000040166	FLRT2	Fibronectin leucine rich transmembrane protein 2	DOWN	Up	Up	UP
ENSSSCG00000039025	FREM1	Fras1 related extracellular matrix 1	DOWN	Up	Up	UP
ENSSSCG00000009364	FREM2	Fras1 related extracellular matrix 2	DOWN	Up	Up	UP
ENSSSCG00000014920	FZD4	Frizzled class receptor 4	DOWN	Up	Up	UP
ENSSSCG00000016430	GALNT11	Polypeptide n-acetylgalactosaminyltransferase 11	DOWN	Up	Up	UP
ENSSSCG00000032241	GPNMB	Glycoprotein nmb	DOWN	Up	Up	UP
ENSSSCG00000039318	HS3ST3B1	Heparan sulfate-glucosamine 3-sulfotransferase 3b1	DOWN	Up	Up	UP
ENSSSCG00000004475	HTR1B	5-hydroxytryptamine receptor 1b	DOWN	Up	Up	UP
ENSSSCG00000026449	KDM4A	Lysine demethylase 4a	DOWN	Up	Up	UP
ENSSSCG00000015555	LAMC1	Laminin subunit gamma 1	DOWN	Up	Up	UP
ENSSSCG00000007430	NEURL2	Neuralized e3 ubiquitin protein ligase 2	DOWN	Down	Down	UP
ENSSSCG00000006236	NSMAF	Neutral sphingomyelinase activation associated factor	DOWN	Down	Up	UP
ENSSSCG00000022676	OPHN1	Oligophrenin 1	DOWN	Down	Up	UP
ENSSSCG00000005665	PHYHD1	Phytanoyl-coa dioxygenase domain containing 1	DOWN	Down	Down	UP
ENSSSCG00000013276	PRDM11	Pr/set domain 11	DOWN	Up	Up	UP
ENSSSCG00000014224	SEMA6A	Semaphorin 6a	DOWN	Up	Up	UP
ENSSSCG00000005177	SH3GL2	Sh3 domain containing grb2 like 2, endophilin a1	DOWN	Up	Up	UP
ENSSSCG00000029815	SRGAP1	Slit-robo rho gtpase activating protein 1	DOWN	Up	Up	UP
ENSSSCG00000015073	TAGLN	Transgelin	DOWN	Up	Up	UP
ENSSSCG00000016457	TAS2R41	Taste 2 receptor member 41	DOWN	Down	Down	UP
ENSSSCG00000030018	ZNF396	Zinc finger protein 396	DOWN	Down	Down	UP
ENSSSCG00000003129	ZSWIM9	Zinc finger swim-type containing 9	DOWN	Up	Up	UP

KEGG pathway enrichment analysis was performed for the candidate DEGs identified in the four pairwise comparisons to explore the biological processes potentially affected by different treatments (Figures 7A–H). Figures 7A–D show the selected KEGG pathways associated with the biological processes of interest, whereas Figures 7E–H integrate Sankey diagrams and bubble plots to illustrate the relationships between DEGs and representative pathways. The identified pathways were mainly distributed across five KEGG categories, including environmental information processing, metabolism, cellular processes, organismal systems, and human diseases, with distinct enrichment patterns among the four comparisons. In the CON vs. FA comparison, nitrogen metabolism showed the most prominent enrichment signal, together with pathways related to IL-17, MAPK, HIF-1, JAK–STAT, and PI3K-Akt signaling. In the CON vs. LPS comparison, the enrichment profile was mainly associated with inflammatory and metabolic responses, including cytokine–cytokine receptor interaction, IL-17 signaling, retinol metabolism, arachidonic acid metabolism, MAPK signaling, and NF-κB signaling. In the CON vs. LPS + FA comparison, TNF signaling showed the strongest enrichment signal, accompanied by NOD-like receptor, IL-17, Toll-like receptor, and other inflammation-related pathways. Ferroptosis, arachidonic acid metabolism, NF-κB signaling, and PI3K-Akt signaling were also included among the biologically relevant pathways selected for exploratory analysis. In the LPS vs. LPS + FA comparison, prominent enrichment was observed in efferocytosis, neuroactive ligand–receptor interaction, calcium signaling, and cytokine–cytokine receptor interaction, while NF-κB, MAPK, PI3K-Akt, and arachidonic acid metabolism were selected as additional pathways of biological interest. Together, these enrichment patterns suggest that FA treatment may be associated with coordinated alterations in inflammatory signaling, lipid metabolism, oxidative stress, and ferroptosis-related processes under LPS challenge.

**Figure 7:**
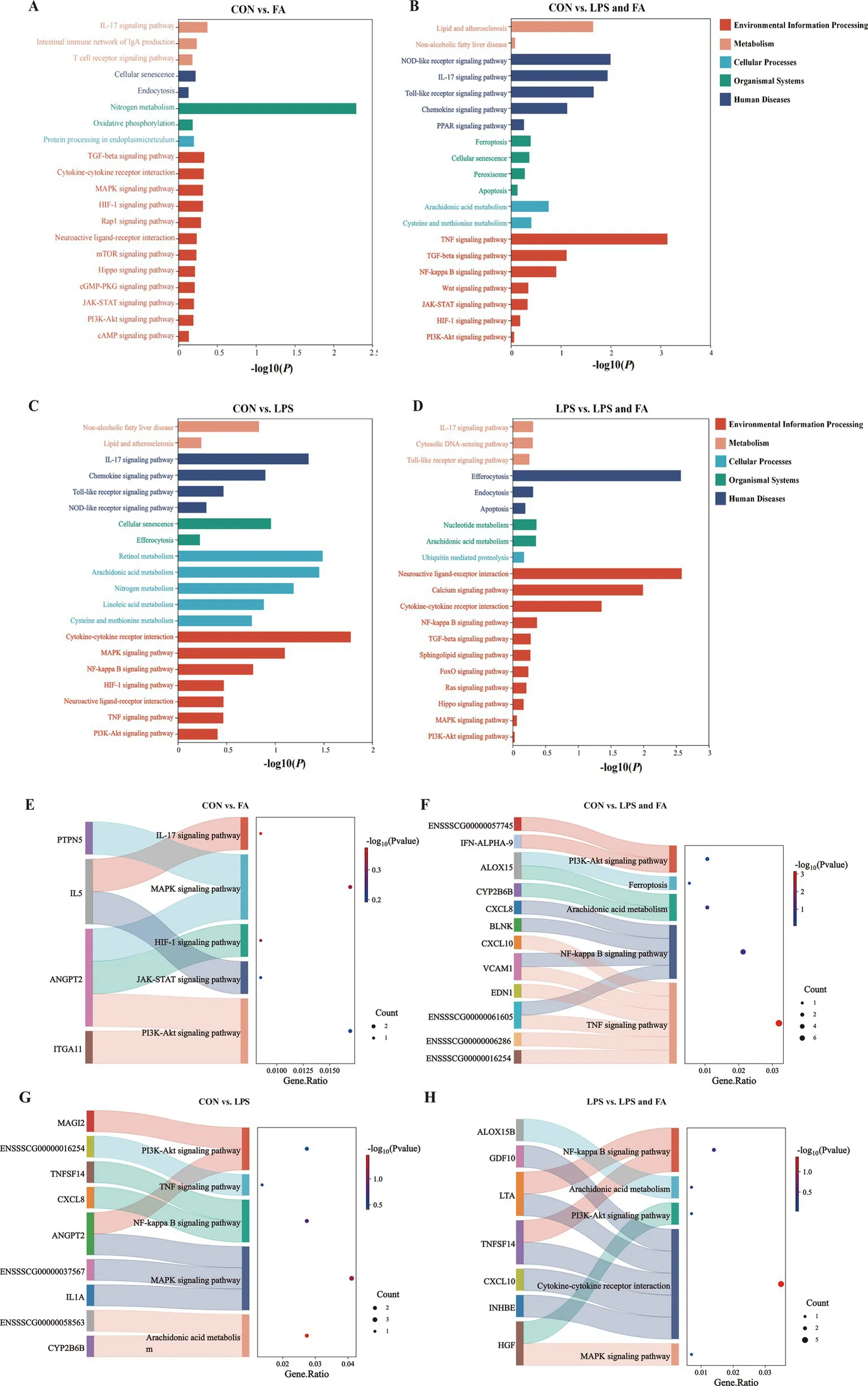
Functional annotation and pathway enrichment analysis. **(A–D)** KEGG pathway enrichment analysis of DEGs in four different comparisons, and **(E–H)** show the sankey diagrams of five key pathways in each group.

For the DEGs in the four comparisons, KEGG pathway enrichment analysis was performed to identify altered biological pathways under different treatments (Figures 7A–H). Figures 7E–H employ a combination of Sankey diagrams and bubble plots to illustrate the relationships between differentially expressed genes and significantly enriched key signaling pathways across comparisons of different treatment groups. All DEGs were mainly classified into five categories: environmental information processing, metabolism, cellular processes, organismal systems, and human diseases, and showed distinct enrichment patterns. DEGs in the CON vs. FA group were enriched in Nitrogen metabolism, MAPK, and JAK–STAT signaling pathways. DEGs in the LPS vs. FA and LPS group were mainly enriched in Efferocytosis, Neuroactive ligand-receptor interaction, Calcium signaling pathway, Cytokine-cytokine receptor interaction, Endocytosis, Apoptosis, Arachidonic acid metabolism, and Nucleotide metabolism. In the CON vs. LPS and CON vs. FA and LPS groups, which involved LPS stimulation, DEGs were widely enriched in key inflammation- and stress-related pathways, including TNF, NF-κB, and HIF-1 signaling pathways. Notably, in the CON vs. FA and LPS group, DEGs were enriched in the Ferroptosis pathway. In addition, DEGs in all four comparisons were enriched in IL-17 and PI3K-Akt signaling pathways.

To further investigate the interactions among DEGs, a protein–protein interaction (PPI) network was constructed based on the DEGs between the CON and LPS groups. As shown in Figure 8A, the network contained 152 nodes and 343 interaction pairs. Among them, genes such as *UBB*, *HMGCS1*, *IL-6*, and *CXCL8* showed high connectivity. These hub genes may coordinately regulate key processes of ferroptosis. *UBB* may affect the stability of proteins such as *GPX4* through the ubiquitin–proteasome system. *HMGCS1* is a key enzyme in the mevalonate pathway, which provides essential metabolic support for maintaining *GPX4* activity. *IL-6* and *CXCL8* are major pro-inflammatory factors. They can amplify oxidative stress and form a vicious cycle between inflammation and oxidation, thereby promoting lipid peroxidation and ferroptosis. A total of 30 hub genes with the highest connectivity were identified using cytoHubba. The top 15 hub genes formed two independent networks. These corresponded to the top two significant modules identified by MCODE, which included 9 nodes with 32 edges and 7 nodes with 21 edges, respectively (Figure 8B). These highly interconnected modules may represent subnetworks that jointly regulate cell death under LPS stimulation. To explore how FA modulates gene interactions, a PPI network was constructed based on 232 co-expressed DEGs shared between the CON vs. LPS and LPS vs. FA and LPS groups. A core interaction network was obtained. *HMGCS1* showed interactions with multiple genes, including *HMGCR*, *CXCL8*, and *IL18* (Figure 8C). These results suggest that FA may reshape the network by regulating the mevalonate pathway axis (HMGCS1/HMGCR) and inhibiting key inflammatory nodes (CXCL8/IL18), thereby coordinately suppressing ferroptosis.

**Figure 8:**
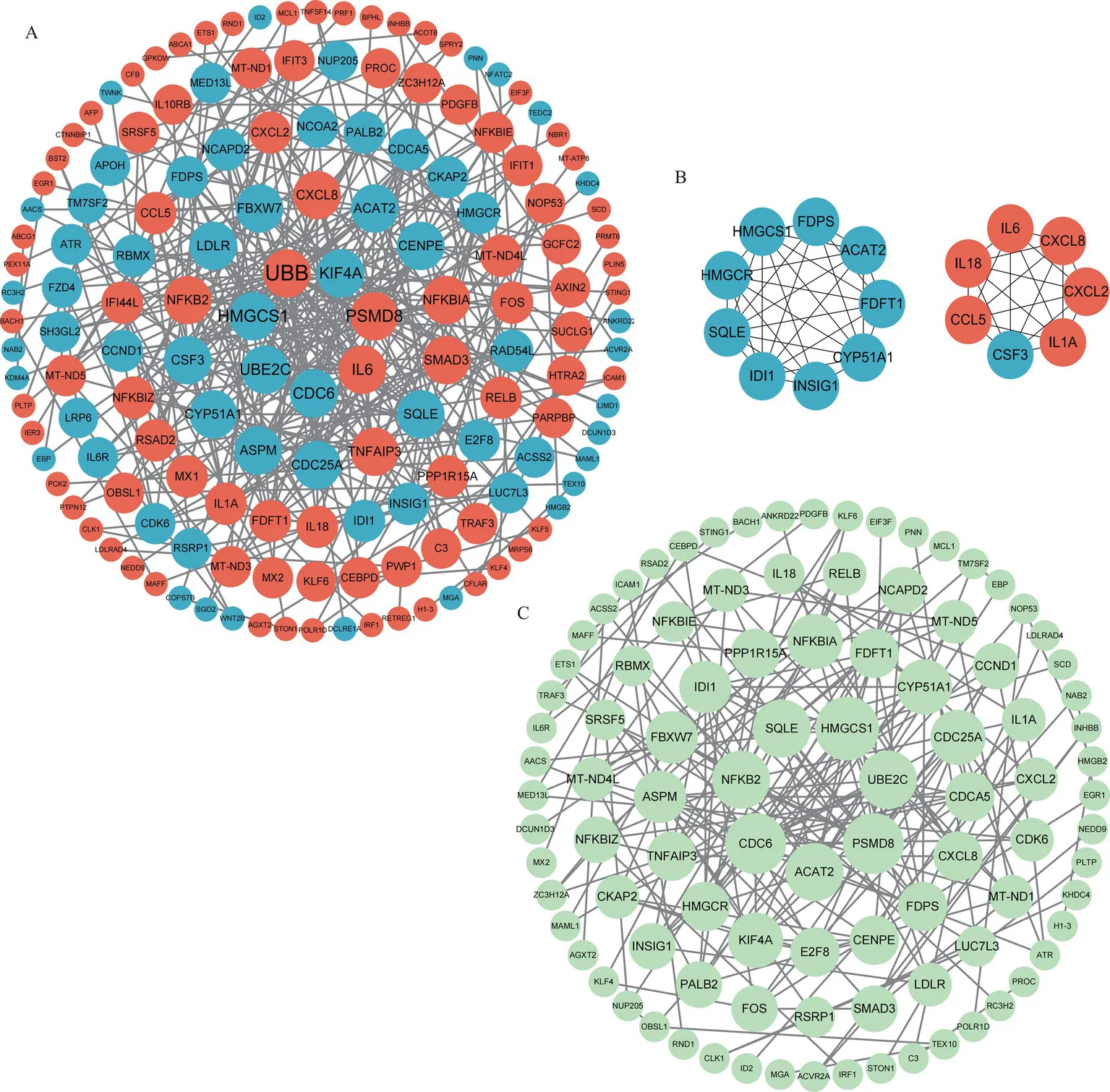
Construction of protein–protein interaction (PPI) networks of DEGs. **(A)** PPI network of DEGs between the CON group and LPS group; **(B)** the top two significant modules extracted from the top 30 DEGs with the highest connectivity in the PPI network (MCODE scores of 9 and 7, respectively); **(C)** PPI network constructed based on 232 co-expressed genes between the CON vs. LPS and LPS vs. FA and LPS groups. Red indicates upregulated genes in **A,B**, and blue indicates downregulated genes. Node size represents the number of interacting genes in **A,C**. The interaction score threshold was set at 0.4 in **A–C**.

To verify the reliability of the transcriptomic sequencing data, eight randomly selected key genes identified from transcriptome analysis were validated via RT-qPCR. As shown in Figure 9, compared with the LPS group, the LPS and FA group significantly upregulated the relative mRNA expression levels of *ABCG1*, *CXCL8*, *LPCAT3*, *IL-6*, *PFKFB3*, and *SCD* (*p* < 0.05). The LPS and FA group also significantly upregulated the relative mRNA expression levels of *ALOX5AP* and *TAGLN* (*p* < 0.05), which was consistent with the transcriptomic results. These findings indicate that the RT-qPCR validation results are consistent with the RNA sequencing data, confirming the reliability and validity of the transcriptomic analysis.

**Figure 9:**
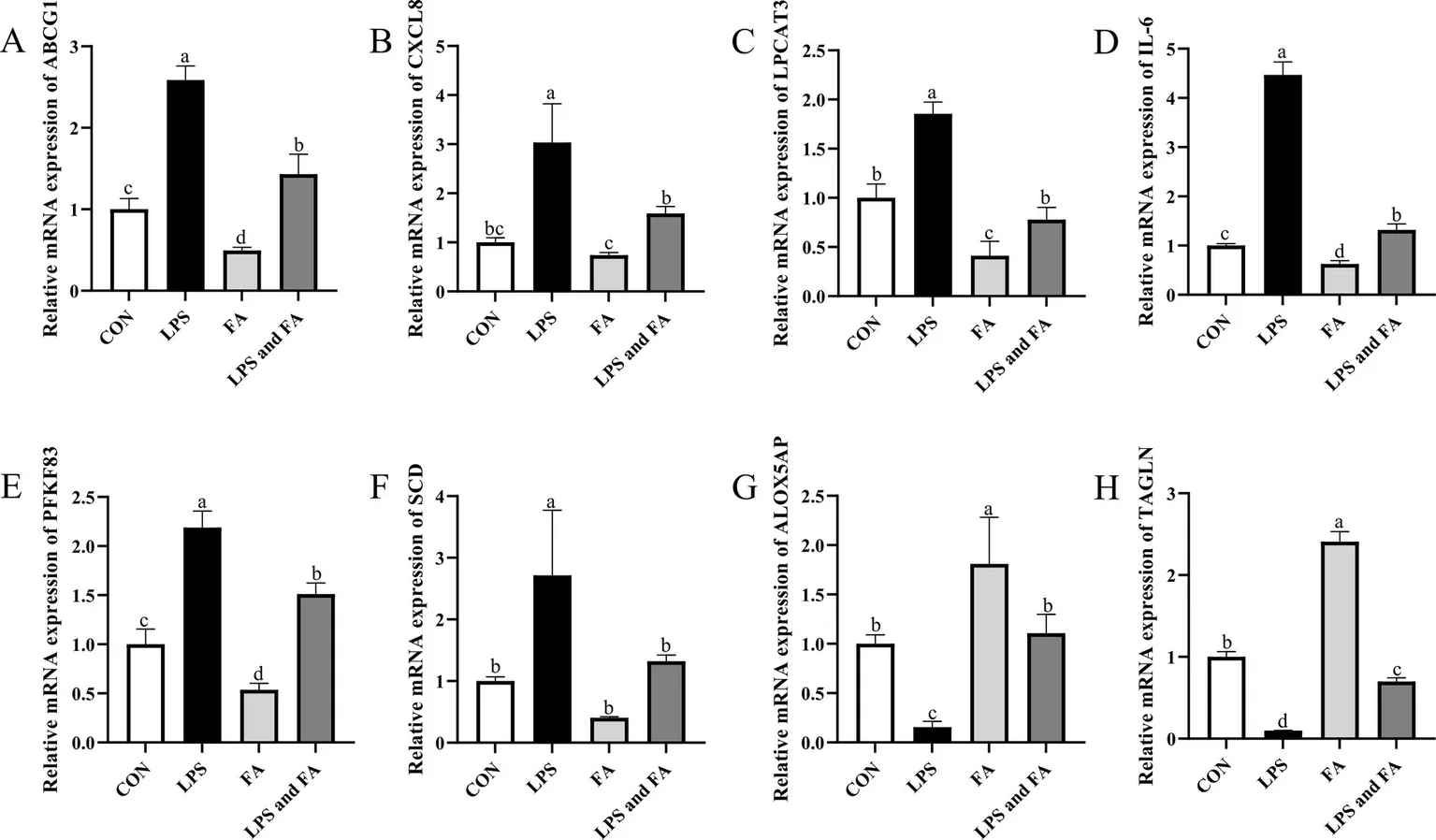
Validation of RNA-seq results by RT-qPCR. **(A–H)** Effects of different treatments on the relative mRNA expression levels of *ABCG1*, *CXCL8*, *LPCAT3*, *IL-6*, *PFKF83*, *SCD*, *ALOX5AP*, and *TAGLN* in IPEC-J2 cells. CON group: control group; LPS group: 10 μg/mL LPS; FA group: 50 μg/mL FA; LPS and FA group: 10 μg/mL LPS + 50 μg/mL FA.

## 4 Discussion

LPS from Gram-negative bacteria causes intestinal epithelial damage in livestock and poultry, but information regarding LPS-triggered ferroptosis in intestinal epithelial cells is scarce (14–16). FA has been reported to possess potent antioxidant and intestinal-protective properties, suggesting its potential application in improving intestinal health. (17, 18, 44) Therefore, this study employed biochemical assays and RNA sequencing to investigate whether and how FA alleviates LPS-induced ferroptosis in IPEC-J2 cells, providing a theoretical basis for the utilization of FA as a functional feed additive. The present study verified that FA inhibits LPS-induced ferroptosis in IPEC-J2 cells. LPS gave rise to canonical ferroptotic characteristics, manifested by decreased cell viability, increased ROS, MDA and Fe^2+^ levels, decreased *GPX4*/*SLC7A11* expression and increased *TFRC*/*ACSL4* expression. FA exhibited protective effects comparable to Fer-1 against LPS-evoked ferroptosis. Transcriptomic analysis suggested that FA partially reversed LPS-induced transcriptional alterations, with candidate DEGs associated with several biologically relevant pathways, including ferroptosis, TNF, NF-κB, arachidonic acid metabolism, and PI3K-Akt signaling. PPI analysis highlighted *HMGCS1*, *CXCL8*, and *IL-18* as potential hub genes. In brief, FA alleviates LPS-triggered ferroptosis by regulating glutathione metabolism, iron homeostasis and lipid peroxidation, which is closely associated with the joint regulation of inflammation, lipid metabolism and ferroptosis-related signaling.

### 4.1 LPS induces ferroptosis in IPEC-J2 cells through iron metabolism imbalance and lipid peroxidation

MDA is a characteristic metabolite of lipid peroxidation, while Fe^2+^ imbalance constitutes a fundamental prerequisite for the occurrence of ferroptosis, and reactive ROS serve as the core mediators driving the ferroptotic process (19–21). Previous studies have demonstrated that LPS can induce a pronounced ferroptotic phenotype in BEAS-2B cells, characterized by Fe^2+^ accumulation and elevated MDA levels (22). These findings are consistent with the results of the present study, in which LPS treatment significantly reduced the viability of IPEC-J2 cells and increased ROS relative fluorescence intensity, as well as MDA and Fe^2+^ contents. Fer-1 is a classical inhibitor of ferroptosis (23). In this study, the addition of Fer-1 effectively reversed the pathological changes in the above indicators, suggesting that LPS successfully induced ferroptosis in IPEC-J2 cells. This is consistent with the findings of Pengfei L (24), who reported that Fer-1 can effectively alleviate LPS-induced ferroptosis in BEAS-2B cells. To elucidate the potential regulatory network underlying LPS-induced ferroptosis in IPEC-J2 cells, transcriptomic analysis was performed in this study. A total of 402 DEGs were identified between the CON and LPS groups. KEGG pathway analysis indicated that these candidate DEGs were associated with several biologically relevant pathways, including TNF, NF-κB, arachidonic acid metabolism, MAPK, and PI3K-Akt signaling pathways, consistent with previous studies (25). Activation of the TNF/NF-κB pathway drives a sustained inflammatory cascade (14). The arachidonic acid metabolism pathway generates key lipid precursors for ferroptosis. Abnormal metabolism in this pathway directly promotes lipid peroxidation and thereby regulates the progression of ferroptosis (26). The MAPK signaling pathway responds to oxidative stress signals. It disrupts intracellular iron homeostasis and ROS balance, thus mediating the initiation and progression of ferroptosis (27). The PI3K-AKT pathway regulates cellular antioxidant capacity and lipid metabolism. In this way, it bidirectionally controls cellular sensitivity to ferroptotic stimuli (28). These pathways act together to aggravate ferroptosis. Their concurrent enrichment suggests that LPS-induced ferroptosis in IPEC-J2 cells is not driven by a single factor. Instead, it results from the combined effects of multiple stress-related networks, including inflammation, hypoxia, and oxidative stress. At the transcriptomic level, this further confirms that LPS can induce ferroptosis in IPEC-J2 cells, which is consistent with the findings of Guo J. (29). To further verify this conclusion, ferroptosis-related genes were examined. *GPX4* and *SLC7A11* are anti-ferroptotic genes, whereas *TFRC* and *ACSL4* are pro-ferroptotic genes (30). The results showed that LPS stimulation significantly downregulated the expression of *GPX4* and *SLC7A11*, while significantly upregulated the expression of *TFRC* and *ACSL4*. Notably, the addition of Fer-1 effectively reversed these gene expression changes. These findings further confirm that LPS induces ferroptosis in IPEC-J2 cells.

### 4.2 Ferulic acid protects IPEC-J2 cells against ferroptosis through modulation of oxidative stress and iron metabolism

As a natural phenolic acid, FA is known for its antioxidant properties and has potential to maintain intestinal health (31, 45). In this study, we examined the direct effects of FA on IPEC-J2 cells. The results showed that an appropriate concentration of FA exerted clear protective effects. This protection is closely related to the regulation of key processes involved in ferroptosis. The GSH/GSSG ratio reflects the cellular redox balance. An increased ratio enhances the glutathione-mediated antioxidant system and upregulates GPX4 activity, thereby slowing ferroptosis (32). In this study, an appropriate concentration of FA not only directly scavenged ROS and reduced MDA levels, but also significantly increased the intracellular GSH/GSSG ratio and decreased Fe^2+^ content. SOD removes excess reactive ROS and alleviates oxidative damage, thereby inhibiting ferroptosis (33). CAT degrades toxic hydrogen peroxide and maintains redox homeostasis, effectively blocking ferroptosis progression (34). However, high concentrations of FA (500–1,000 μg/mL) showed inhibitory effects on SOD and CAT activities. This suggests that excessive FA may disrupt normal enzyme function and induce pro-oxidant effects, which is consistent with the findings of Krawczyk M (35). At the molecular level, FA upregulated the expression of *GPX4* and *SLC7A11*, while downregulated *TFRC* and *ACSL4*. This pattern is consistent with the effect of Fer-1, suggesting that FA can systematically reverse ferroptosis. Transcriptomic analysis further supported these findings. A total of 2,799 DEGs were identified in the CON vs. FA comparison. KEGG enrichment showed that these genes were mainly involved in PI3K-AKT, MAPK, and JAK–STAT pathways. These results indicate that an appropriate concentration of FA alone does not damage IPEC-J2 cells. Instead, it enhances cellular homeostasis by regulating stress-related pathways. This is consistent with the findings of Xiangyi M et al. (36), who reported that FA alone can enhance the antioxidant capacity of IPEC-J2 cells. Therefore, the present study indicates that an appropriate concentration of FA can inhibit ferroptosis in IPEC-J2 cells.

### 4.3 Ferulic acid alleviates LPS-induced ferroptosis in IPEC-J2 cells through regulation of the PI3K-AKT pathway and ferroptosis-related genes

To elucidate the mechanism by which FA alleviates LPS-induced ferroptosis in IPEC-J2 cells, key related indicators were examined in this study. The results showed that FA intervention markedly reversed the key pathological changes induced by LPS. It reduced MDA levels and ROS relative fluorescence intensity, indicating effective inhibition of lipid peroxidation. It also increased the GSH/GSSG ratio and enhanced the activities of antioxidant enzymes such as CAT and SOD, suggesting an overall improvement in the cellular endogenous antioxidant defense system. In addition, FA significantly decreased intracellular Fe^2+^ accumulation, thereby weakening the primary driving force of ferroptosis at its source. Notably, the protective effects of FA were similar to those of Fer-1. These results confirm that FA exerts anti-ferroptotic effects by regulating redox balance and iron homeostasis. This finding is consistent with the conclusions of Tugba K and Li N X (37, 38), and further supports the view that phenolic acids can counteract ferroptosis by enhancing antioxidant capacity and modulating iron metabolism. At the gene level, FA significantly upregulated the expression of *SLC7A11*, thereby promoting GSH synthesis. At the same time, it downregulated the expression of *TFRC* and *ACSL4*. Notably, the effects of FA were similar to those of Fer-1, further indicating that FA can alleviate ferroptosis. Studies have shown that the antioxidant L-theanine can effectively restore gene expression imbalance in Erastin-induced HT22 cells. It reverses the upregulation of *TFRC* and *ACSL4*, as well as the downregulation of *SLC7A11*. This is consistent with the findings of the present study (39). Transcriptomic analysis identified the ferroptosis pathway as one of the biologically relevant pathways associated with the candidate DEGs in the CON vs. LPS + FA comparison. DEGs in the LPS vs. FA and LPS group were mainly enriched in pathways such as arachidonic acid metabolism and cytokine–cytokine receptor interaction. Arachidonic acid is a key substrate for lipid peroxidation, and its metabolic dysregulation is a critical driver of ferroptosis (40). FA intervention can reverse abnormalities in this pathway, thereby directly blocking a core step in the ferroptotic process. Although the ferroptosis pathway was significantly enriched in the CON vs. FA + LPS comparison, it was not identified as a significantly enriched pathway in the LPS vs. LPS + FA comparison, which represents the most direct comparison for evaluating the protective effect of FA against LPS-induced ferroptosis. This discrepancy may be attributed to the inherent characteristics of pathway enrichment analysis. KEGG enrichment relies on the coordinated alteration of multiple genes within a predefined pathway and is highly dependent on the number and statistical significance of differentially expressed genes. Therefore, selective regulation of several key ferroptosis-related genes by FA may not be sufficient to achieve pathway-level enrichment significance. Instead, FA may alleviate ferroptosis by precisely modulating critical molecular nodes involved in iron metabolism, lipid peroxidation, and antioxidant defense rather than broadly remodeling the entire ferroptosis pathway. Importantly, the subsequent validation experiments confirmed that FA significantly reversed LPS-induced ferroptosis-related changes, including the regulation of SLC7A11, TFRC, and ACSL4, further supporting the anti-ferroptotic role of FA. Similarly, salvianolic acid has been reported to alleviate ferroptosis in mice by inhibiting lipid peroxidation (41). A total of 70 co-expressed DEGs were identified between the CON vs. LPS and LPS vs. FA and LPS groups. Among them, 29 genes that were downregulated by LPS were significantly upregulated after FA intervention, while 41 genes that were upregulated by LPS showed the opposite trend. These results suggest that these DEGs represent a core set of target genes underlying the protective effects of FA. Moreover, the RT-qPCR validation results were consistent with the transcriptomic data, confirming the reliability and validity of the sequencing analysis. In the PPI network analysis, a core interaction network was constructed based on co-expressed DEGs. Within this network, *HMGCS1* showed close interactions with genes such as *HMGCR*, *CXCL8*, and *IL18*. As a key gene involved in lipid metabolism, downregulating expression of *HMGCS1* can exacerbate lipid peroxidation (42). FA may inhibit ferroptosis by upregulating *HMGCS1* expression. *CXCL8* and *IL18* are important inflammatory factors. Their interaction may help alleviate the promoting effect of inflammation on ferroptosis. This finding is consistent with the results reported by Zhou H T and Liang Y et al. (43, 46). The enrichment of ferroptosis-related pathways in transcriptomic analysis, together with the involvement of the PI3K-Akt pathway, suggests that FA may regulate the expression of key genes such as HMGCS1 via the PI3K-Akt signaling pathway. On one hand, it suppresses TNF/NF-κB-mediated inflammatory responses. On the other hand, it blocks abnormalities in arachidonic acid metabolism and iron accumulation. Ultimately, these effects contribute to protection against ferroptosis.

This study integrated biochemical assays, ferroptosis-related gene validation, transcriptomic analysis, and PPI network analysis to investigate the protective effects of FA against LPS-induced ferroptosis in IPEC-J2 cells. Potential regulatory targets, including the HMGCS1/HMGCR axis and CXCL8/IL18-related inflammatory network, were identified, and eight candidate DEGs were independently validated by RT-qPCR. However, several limitations remain. DEGs were screened using a nominal *p*-value < 0.05 without multiple-testing correction or a predefined fold-change threshold; therefore, the transcriptomic and network results should be considered exploratory. In addition, β-actin was used as a single reference gene without stability validation under LPS/FA treatment, the identified candidate targets lack functional validation, and only a single intervention time point was examined. Future studies using more stringent transcriptomic criteria, validated reference genes, functional experiments, time-course designs, and *in vivo* models are warranted.

## 5 Conclusion

LPS induced ferroptosis-associated injury in IPEC-J2 cells, characterized by oxidative stress, lipid peroxidation, iron accumulation, impaired antioxidant capacity, and altered expression of ferroptosis-related genes. FA supplementation effectively alleviated these changes, and its protective effects were comparable to those of the ferroptosis inhibitor Fer-1. Transcriptomic and network analyses further suggested that inflammatory signaling, lipid metabolism, and ferroptosis-related processes, including NF-κB, PI3K-Akt, arachidonic acid metabolism, and potential regulatory nodes such as UBB and HMGCS1, may be involved in the protective effects of FA. These findings provide new insights into the potential molecular mechanisms by which FA protects intestinal epithelial cells against LPS-induced ferroptosis and provide a preliminary theoretical basis for its potential application in maintaining intestinal health in livestock and poultry. Further *in vivo* and functional studies are required to validate these proposed mechanisms.

## Data Availability

The datasets presented in this study can be found in online repositories. The names of the repository/repositories and accession number(s) can be found at: https://www.ncbi.nlm.nih.gov/, PRJNA1462534.

## Glossary

LPS: Lipopolysaccharide
FA: Ferulic acid
IPEC-J2: Intestinal Porcine Epithelial Cell line-J2
ROS: Reactive Oxygen Species
MDA: Malondialdehyde
Fe^2+^: Ferrous Ion
GPX4: Glutathione Peroxidase 4
SLC7A11: Solute Carrier Family 7 Member 11
TFRC: Transferrin Receptor
ACSL4: Acyl-CoA Synthetase Long Chain Family Member 4
PPI: Protein–Protein Interaction
HMGCS1: 3-Hydroxy-3-Methylglutaryl-CoA Synthase 1
CXCL8: C-X-C Motif Chemokine Ligand 8
IL-8: Interleukin-8
TLR4: Toll-Like Receptor 4
GSH: Glutathione
FBS: Fetal Bovine Serum
PBS: Phosphate-Buffered Saline
Fer-1: Ferrostatin-1
DMSO: Dimethyl Sulfoxide
SOD: Superoxide Dismutase
T-AOC: Total Antioxidant Capacity
CAT: Catalase
GSSG: Glutathione Disulfide
UBB: Ubiquitin B
IL-6: Interleukin-6
HMGCR: 3-Hydroxy-3-Methylglutaryl-CoA Reductase
IL-18: Interleukin-18
ABCG1: ATP-Binding Cassette Subfamily G Member 1
PFKFB3: 6-Phosphofructo-2-Kinase/Fructose-2,6-Bisphosphatase 3
SCD: Stearoyl-CoA Desaturase
ALOX5AP: Arachidonate 5-Lipoxygenase-Activating Protein
TAGLN: Transgelin
