# Supplementary material for: Comparative transcriptomic analysis reveals the protective mechanism of ferulic acid against lipopolysaccharide-induced ferroptosis in IPEC-J2 cells
Source: Front Vet Sci. 2026 Oct 12;13:1950560. doi: 10.3389/fvets.2026.1950560 (PMC13697013; doi:10.3389/fvets.2026.1950560)
Supplement: Supplementary file 1 [file Data_Sheet_1.docx]

Supplementary Material

# 1 Supplementary Figures

## 1.1 Supplementary Figures 1

**Supplementary Figure 1.** Effects of Fer-1 on LPS-induced ferroptosis in IPEC-J2 cells. CON group: control group; LPS group: 10 μg/mL LPS; LPS and Fer-1 2 group: 10 μg/mL LPS + 2 μmol/L Fer-1; LPS and Fer-1 4 group: 10 μg/mL LPS + 4 μmol/L Fer-1; LPS and Fer-1 6 group: 10 μg/mL LPS + 6 μmol/L Fer-1; LPS and Fer-1 8 group: 10 μg/mL LPS + 8 μmol/L Fer-1; LPS and Fer-1 10 group: 10 μg/mL LPS + 10 μmol/L Fer-1. Bars with the same letters or without letters indicate no significant difference (*p* > 0.05), whereas bars with different lowercase letters indicate significant differences (*p* < 0.05).

## 1.2 Supplementary Figures 2

**Supplementary Figure 2.** Differentially expressed gene (DEG) profiling based on RNA-seq analysis. A Principal component analysis (PCA) of relationships among the 12 samples; B hierarchical clustering and heatmap of DEGs. CON group: control group; FA group: 50 μg/mL FA; FA and LPS group: 10 μg/mL LPS + 50 μg/mL FA; LPS group: 10 μg/mL LPS.
